# Midline defect with corpus callosum agenesis, vermian hypoplasia and median cleft lip palate

**DOI:** 10.1515/crpm-2024-0048

**Published:** 2025-05-05

**Authors:** Clara Illi, Josefine Theresia Koenigbauer, Alexander Weichert

**Affiliations:** Department of Obstetrics, Charité University Hospital, Berlin, Germany; Prenatal Diagnosis and Women’s Health Bergmannstraße, Berlin, Germany

**Keywords:** corpus callosum agenesis, vermian hypoplasia, median cleft lip/palate, syndactyly

## Abstract

**Objectives:**

Midline defects in the brain may be related to genetic syndromes. Association with facial anomalies and skeletal deformities has been described.

**Case presentation:**

In the present case, a routine second trimester scan revealed cerebral abnormalities (corpus callosum agenesis, cerebellar cleft due to vermian hypoplasia, ventriculomegaly), suspected cortical developmental disorder, hypertelorism, a hypoplastic nasal bone, a small median cleft lip and palate, abnormal facial profile, as well as syndactyly of the left hand involving the fourth and fifth finger. Genetic testing revealed a normal karyotype. Subsequent trio exome sequencing did not identify any pathogenic variants or variants of unknown significance. The vaginal delivery at term and postnatal adaptation were uneventful. Postnatal neurosonographic imaging and clinical evaluation confirmed the prenatal findings. Both mother and child were discharged in healthy condition with scheduled follow-ups. Differential diagnoses of the present anomalies include Hartsfield-Bixler-Demyer Syndrome, Oro-Facial-Digital-Syndromes, Ectrodactyly Ectodermal Dysplasia Cleft Lip/Palate Syndrome and Acrocallosal Syndrome.

**Conclusions:**

Invasive diagnostic and genetic testing are recommended when multiple fetal anomalies suggest a potential genetic syndrome. While not all cases reveal an underlying genetic cause, prenatal findings can provide valuable information to help parents and healthcare providers make informed decisions about the continuation of the pregnancy.

## What is already known about this topic?

– Midline defects are rare and may be associated with genetic syndromes.

## What does this study add?

– We report prenatal findings of a midline defect with corpus callosum agenesis, vermian hypoplasia and median cleft lip palate with a normal karyotype and no pathological finding in trio exome sequencing.
– Genetic testing can help parents and healthcare providers with informed decision making regarding affected pregnancy. Still, some fetal anomalies may be unsolved.

## Introduction

Prenatal diagnosis of fetal anomalies regularly confronts healthcare providers and parents with critical questions regarding further procedures and invasive diagnostic. Genetic testing can help with informed decision-making regarding affected pregnancy.

## Case presentation

**Figure 1:**
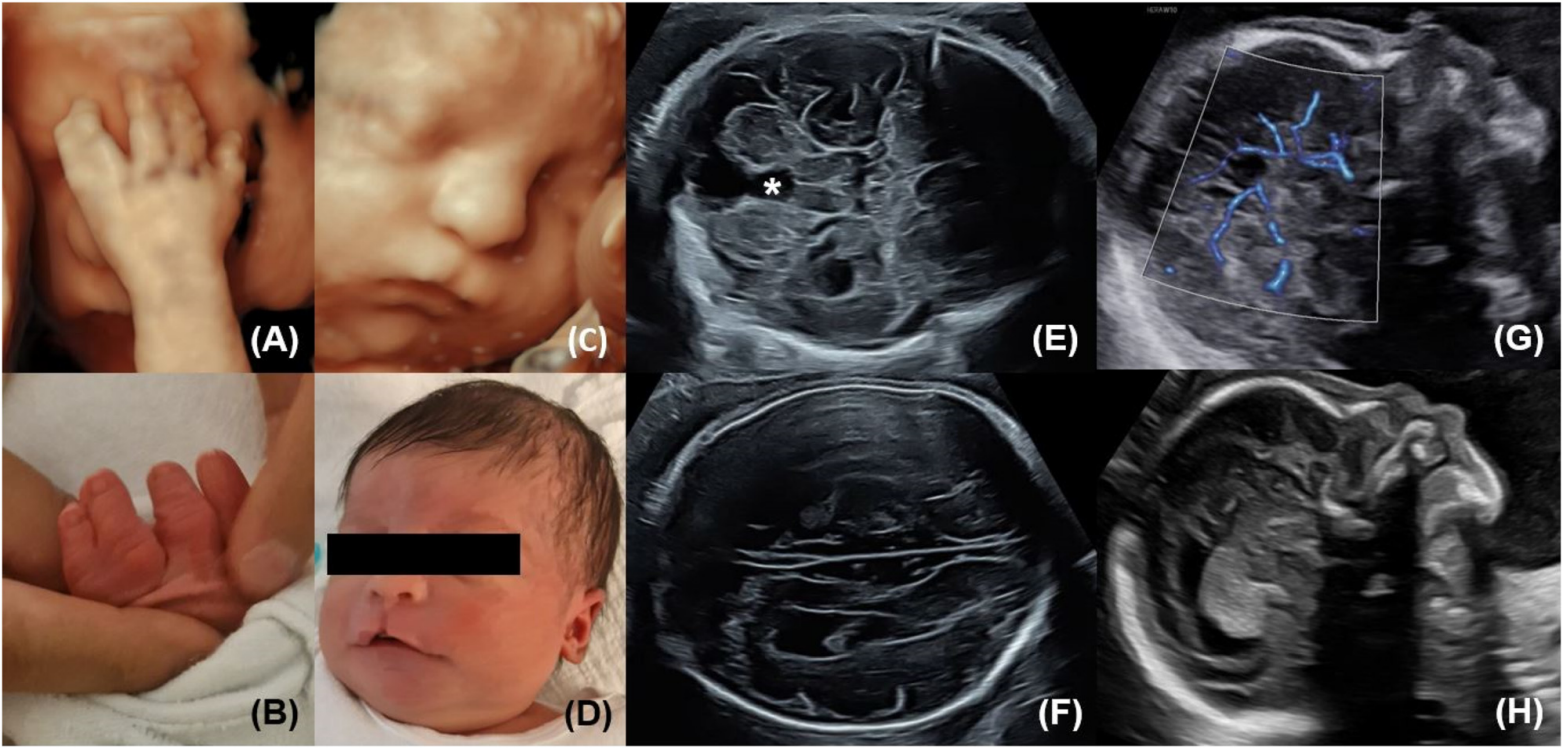
Fetal phenotype: 3D images (A, C), B-mode images (E–H), postnatal images (B, D). (A, B) Syndactyly of the left hand involving fourth and fifth finger; short fingers. (C, D) Small median cleft lip and palate. (E) Cerebellar cleft due to vermian hypoplasia (*). (F–H) Corpus callosum agenesis.

A 24-year-old woman, Gravida II Para I, presented at 21 + 6 gestational weeks for a routine second anomaly trimester scan. The fetal phenotype included a female fetus with corpus callosum agenesis, cerebellar cleft due to vermian hypoplasia, ventriculomegaly, suspected cortical developmental disorder, hypertelorism, a small median cleft lip and palate, as well as syndactyly of the left hand involving the fourth and fifth finger (Figure 1). Fetal and maternal Doppler indices were normal. After genetic counseling the parents opted for further genetic testing and an amniocentesis was performed resulting in a normal karyotype. Trio exome sequencing did not reveal any pathogenic variant or variant of unknown significance. Trio exome sequencing is a method of sequencing the exomes of the affected individual and both biological parents to identify potential disease-causing variants. By comparing the exomes of the affected individual with those of the parents, it helps pinpoint inherited genetic mutations and de novo variants that may contribute to the condition. The family received intensive consultations with pediatric neurologists. A fetal MRI was offered but not favored by the parents. Regular growth scans were normal. The child was delivered vaginally at term without any complications. The sonographic findings were confirmed postnatally. In addition, short fingers were noted (Figure 1). The postnatal adaption was physiological except for some feeding problems. Child and mother could be discharged home after one day.

## Discussion

If the above-mentioned fetal phenotype is detected on prenatal ultrasound and standard cytogenomic testing is non-diagnostic, trio exome sequencing is indicated.

The differential diagnoses include Hartsfield-Bixler-Demyer Syndrome, Oro-Facial-Digital-Syndromes, Ectrodactyly Ectodermal Dysplasia Cleft Lip/Palate Syndrome and Acrocallosal Syndrome. All mentioned syndromes present a wide range of clinical variability.

Hartsfield-Bixler-Demyer Syndrome, also known as fibroblast growth factor 1 (FGFR1)-related Hartsfield Syndrome, is a rare genetic disorder characterized by a combination of holoprosencephaly and ectrodactyly spectrum disorder, as well as facial anomalies including cleft lip and palate [1]. Other associated midline brain malformations include corpus callosum agenesis, absent septum pellucidum and vermian hypoplasia.

The Oro-Facial-Digital Syndromes include at least 13 genetically heterogeneous disorders characterized by anomalies of the oral cavity, facial features and digits. Additional abnormalities in the brain and visceral organs have been described [2].

Ectrodactyly-Ectodermal Dysplasia-Cleft Lip/Palate Syndrome is a rare genetic disorder characterized by ectrodactyly, ectodermal dysplasia and cleft lip/palate. It is an autosomal dominant condition primarily associated with variations in the tumor protein 63 (TP63) gene [3].

Acrocallosal Syndrome is an autosomal recessive disorder caused by variants in the kinesin family member 7 (KIF7) gene. It is characterized by agenesis of the corpus callosum, midline brain and facial abnormalities, polydactyly, and in most cases psychomotor developmental delay [4].

Corpus callosum agenesis is one of the most frequent brain malformations and involves congenital absence of all or parts of the corpus callosum. It appears as an isolated condition or in association with a genetic syndrome. A genetic cause can be identified in 12.5 % with chromosomal microarray and up to 47 % with whole exome sequencing [5]. The postnatal outcome varies considerably, even in isolated corpus callosum agenesis outcomes may range from normal in 65 % to mild to severe neurodevelopmental impairments in 35 % [6].

The present case shows that even highly pathologic sonographic findings are not necessarily associated with pathologic genetic findings in trio exome sequencing or a specific genetic syndrome.

In conclusion, genetic as well as specific pediatric counseling is recommended when anomalies are suspected to discuss the nature of the disorder, inheritance patterns, and implications for the pregnancy and family planning.
